# In situ follicular neoplasia in a young post‐liver transplant patient

**DOI:** 10.1111/pin.13291

**Published:** 2022-12-12

**Authors:** Rachel Dobson, Lakshmi Venkatraman, Francesco Cucco, Zi Chen, Jennifer Jones, Michael Quinn, Ming‐Qing Du

**Affiliations:** ^1^ Department of Pathology, Division of Cellular and Molecular Pathology University of Cambridge Cambridge UK; ^2^ Royal Victoria Hospital, Belfast Health and Social Care Trust Belfast UK; ^3^ Cambridge Genomics Laboratory Cambridge University Hospitals NHS Foundation Trust Cambridge UK; ^4^ Belfast City Hospital, Belfast Health and Social Care Trust Belfast UK; ^5^ Department of Histopathology, Addenbrooke's Hospital Cambridge University Hospitals NHS Foundation Trust Cambridge UK

**Keywords:** in situ follicular neoplasia, mutation, liver transplantation

**Abbreviations**
ISFN: in situ follicular neoplasia
LN: Lymph nodes
PET‐CT: positron emission tomography computed tomograph
PTLD: post‐transplant lymphoproliferative disorder
PCR: polymerase chain reaction
TNFRSF14: TNF Receptor Superfamily Member 14

To the Editor,

T(14:18)(q32;q21), causing *IGH::BCL2*, occurs as a result of erroneous genomic rearrangements during VDJ recombination at the pre‐B stage of B‐cell development in the bone marrow. The translocation can be detected in peripheral blood lymphocytes in healthy individuals by polymerase chain reaction (PCR) and its prevalence depends on age, ranging from 0% in individuals <10 years old to 60% in those >40 years age. These circulating translocation positive cells are thought to correspond to those of in situ follicular neoplasia (ISFN) (in situ follicular B‐cell neoplasm according to the 5th edition of the World Health Organization classification of haematolymphoid tumours) in lymph nodes (LN), which are incidentally identified by their strong BCL2 expression by immunohistochemistry. Besides the translocation, several pathogenic mutations have also been described in ISFN, albeit exclusively from patients >40 year olds to date.^1^, ^2^, ^3^ It is unclear when such mutations occur during the clonal expansion of *IGH::BCL2* positive B‐cells, whether the mutations occur in young patients? Herein, we report genetic analyses of ISFN in a young patient.

The patient is a 24 year old male with a history of Crohn's disease and autoimmune hepatitis. He received two liver allografts at age 14 and 21 years, and was evaluated for a third transplant due to liver graft failure. Non‐bulky periportal, mesenteric lymphadenopathy, and splenomegaly were identified on computed tomograph (CT) scan in 2017. Core needle biopsies from a prehepatic LN showed reactive hyperplasia with no evidence of lymphoma. Hematological review demonstrated no disease progression. Subsequently, a mild generalized lymphadenopathy was identified on positron emission tomography (PET) CT with a whole right groin LN excised in January 2021.

The groin LN showed preservation of the follicular and sinus architecture. There was an increase in germinal centers with interfollicular and perifollicular reactive polytypic plasmacytosis. Many (~40%) of the follicles showed a monotonous population of centrocytes with few centroblasts and absence of tingible body macrophages, and displayed both strong CD10 and BCL2 staining (Figure 1a). The morphology and immunophenotype suggested ISFN and there was no evidence of an overt lymphoma or PTLD (posttransplant lymphoproliferative disorder) though presence of occasional EBV‐positive cells in the interfollicular areas was noted by EBER‐in situ hybridization. Analysis of whole tissue section of the LN by BIOMED‐2 clonality assays showed no evidence of clonal *IG* gene rearrangements.

**Figure 1:**
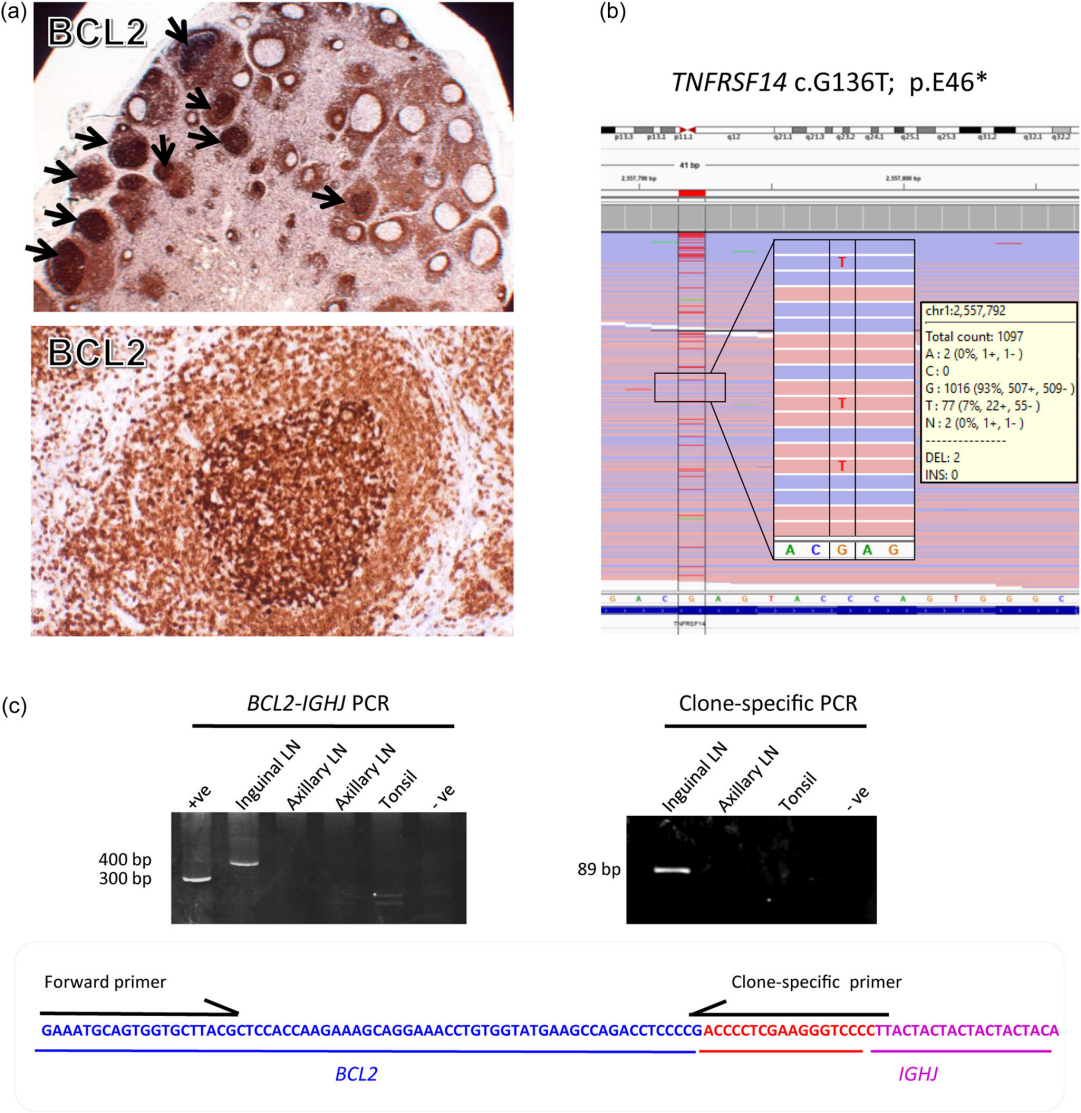
Genetic characterization of in situ follicular neoplasia (ISFN) in a young liver‐transplant patient. (a) Identification of ISFN by BCL2 immunohistochemistry. ISFN lesions indicated by arrows. (b) Targeted sequencing of 191 genes (frequently mutated in follicular lymphoma and diffuse large B‐cell lymphoma) identifies a single mutation, namely *TNFRSF14* c.G136T, p.E46*, which predicts a truncated protein product. (c) *BCL2‐IGHJ* PCR (MBR1) (BIOMED‐2 Reaction A assay) shows a positive product for the inguinal lymph node (LN), but not the axillary LN biopsy (left panel). Clone‐specific PCR demonstrates an expected 89 bp product from the inguinal LN biopsy, but not the axillary LN biopsy (right panel). Nucleotides in red color (bottom panel) are nontemplate random insertions between the *BCL2* and *IGHJ* genes.

Flow cytometry analysis of peripheral blood samples showed no evidence of clonal B‐cells. Bone marrow aspirate and trephine examination was negative for malignancy. PCR analysis of the peripheral blood mononuclear cells for EBV was positive, and follow‐up analyses showed decline of EBV load. Six months later, a follow‐up positron emission tomography computed tomograph revealed avid right axillary LN, which was excised and shown to be histologically reactive, without any evidence of ISFN by BCL2 immunohistochemistry. The patient underwent an orthotropic liver transplant in November 2021, and was well apart from wound dehiscence and mild pancytopenia following the transplant surgery.

The ISFN lesions in the right groin LN were microdissected for DNA extraction and was subjected to mutation analysis by targeted sequencing of 191 genes (frequently mutated in follicular lymphoma and diffuse large B‐cell lymphoma) using the TWIST target enrichment kit (Supporting Information: Materials and methods).^4^ This revealed a single pathogenic mutation, TNF Receptor Superfamily Member 14 (*TNFRSF14* c.G136T), p.E46* (Figure 1b). Targeted sequencing analysis of the right axillary LN, which showed no evidence of ISFN, did not identify any pathogenic mutations.

We also performed PCR analyses of *BCL2*‐*IGHJ* genomic fusion using the BIOMED‐2 assays, followed by Sanger sequencing (Supporting Information: Figure 1). This identified a *BCL2‐IGHJ* fusion in the right inguinal LN, but not the subsequent right axillary LN biopsy (Figure 1c). To further rule out the presence of *BCL2*‐*IGHJ* positive cells in the axillary LN, we designed a clone‐specific PCR using primers targeting the *BCL2‐IGHJ* junction sequence, and investigated the axillary LN specimen again (Figure 1c). This additional PCR analysis confirmed the absence of the translocation in the axillary LN.

To further rule out the possibility of donor origin of the *BCL2* translocation positive cells, we compared the short tandem repeat (STR) genotype between the ISFN and non‐neoplastic reactive components isolated from the right inguinal LN using the GenomeLab Human STR Primer Set Kit (Beckman Coulter). The results showed no difference in the STR genotype between the two components, indicating that the *BCL2* translocation positive cells originated from the host.

*TNFRSF14* mutation is one of the frequent genetic changes in follicular lymphoma,^4^ and has also been reported in ISFN of patients >40 years old, often concurring with other pathogenic variants.^2^ Unlike the cases reported in the literature, *TNFRSF 14* mutation in the present case is the sole genetic change detected apart from *BCL2* translocation. The mutation (p.E46*) predicts a truncated protein product, thus deleterious to the protein function. TNFRSF14 expression on B‐cells restrains T‐cell help, modulating B‐cell responses during the germinal center reaction.^5^ In mice, Tnfrsf14 (HVEM) deficiency increased B‐cell competitiveness during germinal center responses, conferring them to a proliferation advantage, although insufficient for malignant transformation.^5^ It remains to be investigated to what extent the additional *TNFRSF14* mutation increases the risk of follicular lymphoma development.

The occurrence of ISFN in a young adult, such as this patient, is unusual. *BCL2* translocation is the result of erroneous VDJ recombination, while *TNFRSF14* mutation is most likely the consequence of somatic hypermutation process. Although acquisition of these genetic changes is likely incidental, the clonal expansion of the mutated B‐cells might be influenced by the patient's autoimmune conditions (Crohn's disease and autoimmune hepatitis) and immunosuppression following liver transplant.

## AUTHOR CONTRIBUTIONS

Rachel Dobson performed the majority of experimental works and data analysis for the manuscript. Lakshmi Venkatraman initiated the case study and histological assessment. Francesco Cucco and Zi Chen carried out NGS and related data analysis. Jennifer Jones carried out genotyping analysis. Michael Quinn provided clinical information and input. Ming‐Qing Du organized the study and wrote the manuscript together with Rachel Dobson and Lakshmi Venkatraman. All authors approved the manuscript.

## CONFLICT OF INTEREST

None declared.

## ETHICS STATEMENT

Local ethical guidelines were followed for the use of archival tissues for research with ethical approval (05‐Q1604‐10).

## Supporting information

Supporting information.

Supporting information.

## References

[1] Dobson R , Wotherspoon A , Liu SA , Cucco F , Chen Z , Tang Y , et al. Widespread in situ follicular neoplasia in patients who subsequently developed follicular lymphoma. J Pathol. 2022;256:369–77. 34957565 10.1002/path.5861 PMC9310836

[2] Vogelsberg A , Steinhilber J , Mankel B , Federmann B , Schmidt J , Montes‐Mojarro IA , et al. Genetic evolution of in situ follicular neoplasia to aggressive B‐cell lymphoma of germinal center subtype. Haematologica. 2021;106:2673–81. 32855278 10.3324/haematol.2020.254854 PMC8485666

[3] Schmidt J , Ramis‐Zaldivar JE , Bonzheim I , Steinhilber J , Müller I , Haake A , et al. CREBBP gene mutations are frequently detected in in situ follicular neoplasia. Blood. 2018;132:2687–90. 30401710 10.1182/blood-2018-03-837039 PMC6302496

[4] Cucco F , Barrans S , Sha C , Clipson A , Crouch S , Dobson R , et al. Distinct genetic changes reveal evolutionary history and heterogeneous molecular grade of DLBCL with MYC/BCL2 double‐hit. Leukemia. 2020;34:1329–41. 31844144 10.1038/s41375-019-0691-6 PMC7192846

[5] Mintz MA , Felce JH , Chou MY , Mayya V , Xu Y , Shui JW , et al. The HVEM‐BTLA axis restrains T cell help to germinal center B cells and functions as a cell‐extrinsic suppressor in lymphomagenesis. Immunity. 2019;51:310–23. 31204070 10.1016/j.immuni.2019.05.022 PMC6703922

