# Supplementary material for: In situ follicular neoplasia in a young post‐liver transplant patient
Source: Pathol Int. 2022 Dec 12;73(1):58–60. doi: 10.1111/pin.13291 (PMC10107664; doi:10.1111/pin.13291)
Supplement: Supplementary file 1 — Supporting information. [file PIN-73-58-s001.docx]

**MATERIALS AND METHODS**

**Tissue material and DNA extraction**

Local ethical guidelines were followed for the use of archival tissues for research with the approval of the ethics committee (05-Q1604-10).

Spare tissue sections from two formalin‐fixed paraffin‐embedded (FFPE) lymph node excision biopsies were available for the study. The *in situ* follicular neoplasia (ISFN) lesions were identified by BCL2 immunohistochemistry, and then microdissected manually from consecutive unstained sections. The microdissected ISFN lesions, and uninvolved tissue, where indicated whole tissue section, were subjected to DNA extraction using QIAamp DNA micro-Kit (Qiagen, Crawley, UK). The quality of DNA was assessed by PCR of variably sized genomic fragments (200bp, 300bp, 400bp, 600bp) using a standardized protocol.^1^ Both biopsies showed amplification of up to 600bp of genomic fragments.

**PCR and sequencing of the *BCL2-IGHJ* fusion**

The *BCL2-IGHJ*  genomic fusion was amplified using the BIOMED‐2 method,^2^ using the primers for MBR (reaction A), 3’MBR (reaction B) and mcr (reaction C), which were tagged with a common sequence (CS1: 5'‐ACACTGACGACATGGTTCTACA‐3′, or CS2: 5'‐TACGGTAGCAGAGACTTGGTCT‐3′) to facilitate sequencing. All PCRs were performed in duplicate, using 30-40ng input DNA in 25μl reaction volumes. PCRs were carried out using FastStart High Fidelity PCR system (Roche, Basel, Switzerland) and cycling conditions are detailed in Supplementary Table 1. The amplified products were analyzed by electrophoresis on 10% polyacrylamide gels. The positive PCR products were purified using AMPure XP beads (Beckman Coulter, Pasadena, CA, USA) and then sequenced using the Sanger method (Department of Biochemistry, University of Cambridge sequencing facility). The unique junctional sequence between the *BCL2* and *IGH* gene was identified using BLAT/BLAST analysis ([www.ensembl.org](http://www.ensembl.org)).

**Supplementary Table 1:**  PCR conditions used for amplification of *BCL2-IGHJ* genomic fusion.

| **Temperature** (°C) | **Time** | **cycles** |
| --- | --- | --- |
| 95 | 10 min | x1 |
| 96 | 45 sec | x40 |
| 60 | 45 sec |  |
| 72 | 90 sec |  |
| 72 | 10 min | x1 |
| 25 | 10 min | x1 |

**Clone-specific PCR (CS-PCR)**

Clone-specific primer was designed to bind to the unique *BCL2-IGHJ* junction sequence as outlined in Figure 1C. This, together with a primer targeting the upper-stream *BCL2* sequence formed a strategy for CS-PCR (Figure 1, Supplementary Table 2). CS-PCRs were carried out using FastStart High Fidelity PCR system (Roche) and touchdown cycling conditions (Supplementary Table 2) as previously described.^3^ The sensitivity of the CS-PCR was determined by PCR of 10-fold serial dilutions of the DNA extracted from the inguinal lymph node biopsy (containing ISFN lesions) using tonsil DNA (Supplementary Figure 1D). All PCRs were carried out in duplicate, and the amplified products were analyzed by electrophoresis on 10% polyacrylamide gels. The CS-PCR was used to investigate whether the ISFN clone was present in the subsequent axillary lymph node biopsy.

**Supplementary Table 2:** Summary of clone-specific PCR primers and conditions.

|  |  |  |  |
| --- | --- | --- | --- |
| Forward Primer  (BCL2 sequence) | | 5’ GAAATGCAGTGGTGCTTACGCT 3’ | Amplicon size (bp) |
| Reverse Primer  (BCL2-IGHJ junction sequence) | | 5’ TAAGGGGACCCTTCGAGGGGT 3’ | 89 |

| **Optimized CS-PCR cycle conditions** | | |
| --- | --- | --- |
| Temperature (°C) | Time | No. of Cycles |
| 95 | 10 min | 1 |
| 95 | 30 sec | 10 |
| 67-57 | 30 sec |  |
| 72 | 1 min |  |
| 95 | 30 sec | 38 |
| 57 | 30 sec |  |
| 72 | 1 min |  |
| 72 | 5 min | 1 |

**Mutation analysis by targeted sequencing**

The purified DNA samples from both lymph node biopsies were subjected to targeted sequencing of 191-genes, which were frequently mutated in follicular lymphoma and diffuse large B-cell lymphoma. This was carried out by using TWIST Bioscience target enrichment and Illumina HiSeq sequencing. As the samples were relatively good quality DNA (supporting PCR of 600bp genomic fragment) (Supplementary Figure 1A ), the target sequencing was performed in a single replicate. The sequence data analysis, variant calling, and filtering were performed as described in our previous studies,^1,4^ with 2% variant allele frequency as cut-off.

**Genes investigated by targeted next generation sequencing**

| ABCA10 | CUX1 | HVCN1 | NRAS | TBL1XR1 |
| --- | --- | --- | --- | --- |
| ABCA6 | CXCR4 | IBTK | OSBPL10 | TCF3 |
| ADGRL2 | CYLD | ID3 | P2RY8 | TCF4 |
| ARID1A | DDX10 | IFI44 | PAPOLG | TET2 |
| ARID1B | DDX3X | IGLL5 | PAX5 | TFEB |
| ARID4B | DDX46 | IL10RA | PDCD1LG2 | TFPT |
| ARID5B | DDX5 | ILF3 | PERP | TLR2 |
| ATM | DNMT3A | IRF4 | PIK3CD | TMEM30A |
| B2M | DTX1 | IRF8 | PIM1 | TMSB4X |
| BCAT1 | DUSP2 | ITPKB | PIM2 | TNFAIP3 |
| BCL10 | EBF1 | KLF2 | PLCG2 | TNFRSF14 |
| BCL11A | EP300 | KLHL14 | POU2AF1 | TNFSF13B |
| BCL2 | ERCC5 | KLHL2 | POU2F2 | TNFSF14 |
| BCL6 | ETS1 | KLHL6 | PPM1D | TOX |
| BCL7A | ETV6 | KMT2D | PRDM1 | TP53 |
| BRAF | EZH2 | KRAS | PRDM15 | TRAF3 |
| BTG1 | FAS | LPP | PRKCB | TRRAP |
| BTG2 | FBXW7 | LTB | PTEN | UBE2A |
| BTK | FNDC3A | MALT1 | PTPN6 | WEE1 |
| C2CD2 | FOXO1 | MAP2K1 | PVT1_promoter | WISP1 |
| C4B_2 | FOXO3 | MCL1 | QTRT1 | XPO1 |
| CA8 | FXYD6 | MEF2B | RB1 | ZC3H12A |
| CARD11 | GNA13 | MIB2 | RCOR1 | ZDHHC18 |
| CCND3 | GRB2 | MIR17HG | REL | ZEB2 |
| CD19 | GTDC1 | MLLT3 | RELB | ZFAND5 |
| CD274 | HIST1H1B | MPEG1 | RHOA | ZFHX3 |
| CD58 | HIST1H1C | MTOR | RRAGC | ZFP36L1 |
| CD70 | HIST1H1D | MYC | S1PR2 | ZNF106 |
| CD79A | HIST1H1E | MYC_Promoter/5'UTR | SF3B1 | ZNF292 |
| CD79B | HIST1H2AC | MYD88 | SGK1 | ZNF296 |
| CD83 | HIST1H2AG | NAV1 | SLA | ZNF423 |
| CDC73 | HIST1H2AM | NCKIPSD | SMARCA4 |  |
| CDKN1B | HIST1H2BC | NCOR1 | SOCS1 |  |
| CDKN2A | HIST1H2BK | NFATC1 | SOX5 |  |
| CDKN2B | HIST2H2BE | NFKBIA | SPEN |  |
| CIITA | HLA-A | NFKBIE | SPIB |  |
| COL12A1 | HLA-B | NFKBIZ_3'UTR | STAT3 |  |
| COX10 | HLA-C | NLRP8 | STAT6 |  |
| CREBBP | HNRNPD | NOTCH1_last exon | SUMO2 |  |
| CTDP1 | HNRNPDL | NOTCH2_last exon | SYK |  |

**Short Tandem Repeat (STR) genotyping**

This was carried out by PCR of fifteen STR loci (D18S51, D21S11, TH01, D3S1358, Penta E, FGA, TPOX, D8S1179, vWA, CSF1PO, D16S539, D7S820, D13S317, D5S818 and Penta D) and one sex identification locus (Amelogenin) using the PowerPlex® 16 HS System (Promega). The PCR products were analyzed on an Applied Biosystems Genetic Analyzer.

**REFERENCES:**

1. Cucco F, Clipson A, Kennedy H *et al.* Mutation screening using formalin-fixed paraffin-embedded tissues: a stratified approach according to DNA quality. *Lab Invest.* 2018; **98:** 1084-1092.

2. van Dongen JJ, Langerak AW, Brüggemann M *et al.* Design and standardization of PCR primers and protocols for detection of clonal immunoglobulin and T-cell receptor gene recombinations in suspect lymphoproliferations: report of the BIOMED-2 Concerted Action BMH4-CT98-3936. *Leukemia.* 2003; **17:** 2257-2317.

3. Dobson R, Wotherspoon A, Liu SA *et al.* Widespread in situ follicular neoplasia in patients who subsequently developed follicular lymphoma. *J Pathol.* 2022; **256:** 369-377.

4. Cucco F, Barrans S, Sha C *et al.* Distinct genetic changes reveal evolutionary history and heterogeneous molecular grade of DLBCL with MYC/BCL2 double-hit. *Leukemia.* 2020; **34:** 1329-1341.
