# Supplementary material for: In situ follicular neoplasia in a young post‐liver transplant patient
Source: Pathol Int. 2022 Dec 12;73(1):58–60. doi: 10.1111/pin.13291 (PMC10107664; doi:10.1111/pin.13291)
Supplement: Supplementary file 2 — Supporting information. [file PIN-73-58-s002.pptx]

## Slide 1
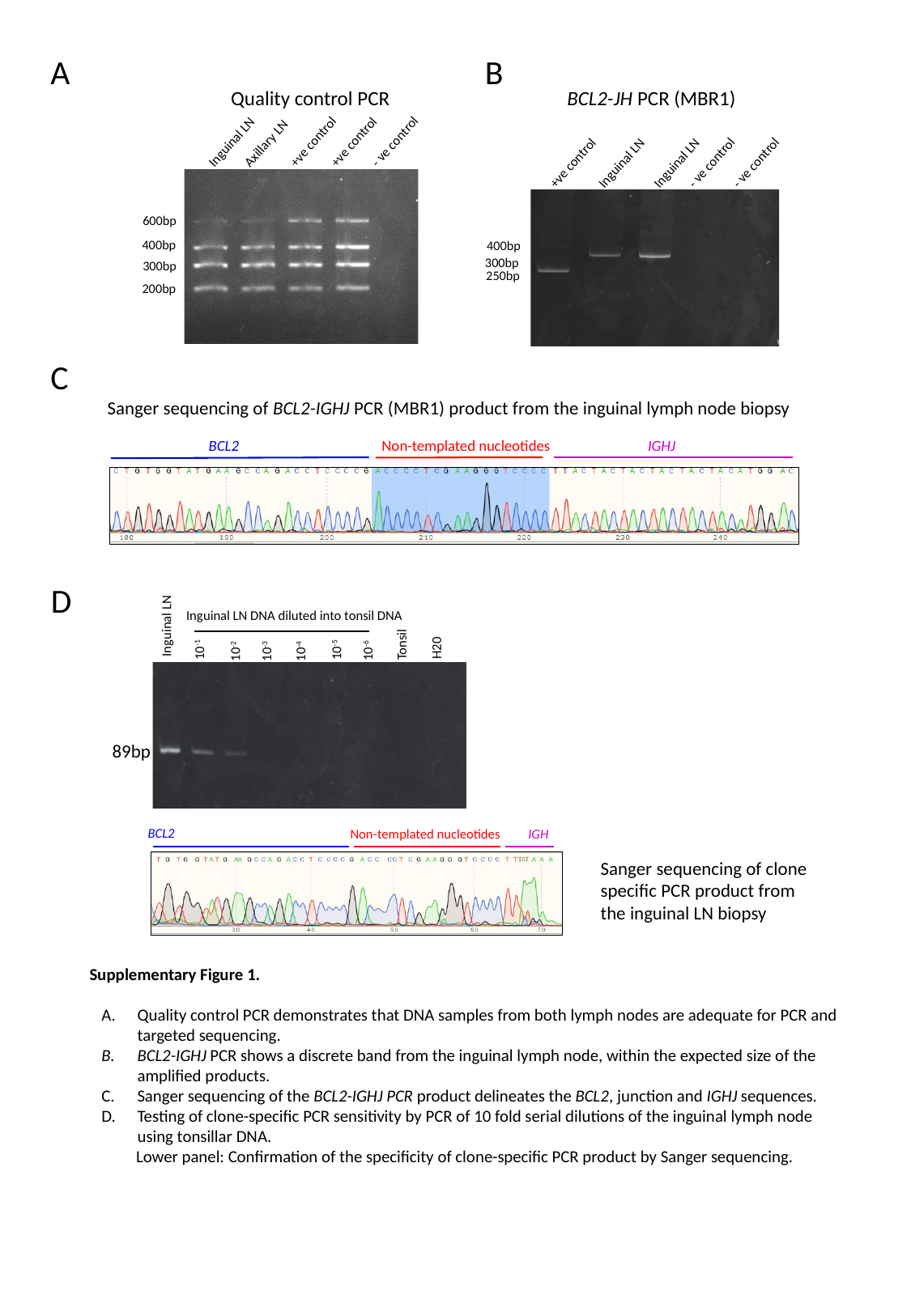

A
B
Quality control PCR
BCL2-JH PCR (MBR1)
Axillary LN
Inguinal LN
+ve control
+ve control
- ve control
Inguinal LN
Inguinal LN
+ve control
- ve control
- ve control
400bp
300bp
250bp
600bp
400bp
300bp
200bp
C
Sanger sequencing of BCL2-IGHJ PCR (MBR1) product from the inguinal lymph node biopsy
BCL2
Non-templated nucleotides
IGHJ
D
Inguinal LN DNA diluted into tonsil DNA
Inguinal LN
H20
10-1
10-5
Tonsil
10-6
10-2
10-3
10-4
89bp
BCL2
IGH
Non-templated nucleotides
Sanger sequencing of clone specific PCR product from the inguinal LN biopsy
Supplementary Figure 1.
Quality control PCR demonstrates that DNA samples from both lymph nodes are adequate for PCR and targeted sequencing.
BCL2-IGHJ PCR shows a discrete band from the inguinal lymph node, within the expected size of the amplified products.
Sanger sequencing of the BCL2-IGHJ PCR product delineates the BCL2, junction and IGHJ sequences.
Testing of clone-specific PCR sensitivity by PCR of 10 fold serial dilutions of the inguinal lymph node using tonsillar DNA.
 Lower panel: Confirmation of the specificity of clone-specific PCR product by Sanger sequencing.
